# Stress Hyperglycemia Is Associated with Unfavorable Outcomes After Mechanical Thrombectomy in Patients with Acute Ischemic Stroke

**DOI:** 10.3390/brainsci15040360

**Published:** 2025-03-30

**Authors:** Jie Gao, Xiangliang Chen, Qing Huang, Mengmeng Gu, Ye Hong, Gelin Xu

**Affiliations:** 1 Department of Neurology, Nanjing Jinling Hospital, Affiliated Hospital of Medical School, Nanjing University, Nanjing 210002, China; gaojienj1992@163.com; 2 Department of Neurology, Nanjing First Hospital, Nanjing Medical University, Nanjing 210006, China; chenxl@njmu.edu.cn (X.C.); huangqing70209@163.com (Q.H.); gumengmeng322@yeah.net (M.G.); dg1735053@smail.nju.edu.cn (Y.H.)

**Keywords:** stress hyperglycemia, ischemic stroke, symptomatic intracranial hemorrhage, early neurological deterioration, post-stroke pneumonia, modified Rankin scale

## Abstract

**Background:** Stress hyperglycemia may deteriorate stroke outcomes, but its impact on the prognosis following mechanical thrombectomy remains unclear. This study aimed to evaluate the effects of stress hyperglycemia on in-hospital and 3-month outcomes in stroke patients with anterior circulation occlusion undergoing mechanical thrombectomy. **Methods:** A total of 415 patients who had mechanical thrombectomy in the anterior circulation were enrolled. The stress hyperglycemia ratio (SHR) was calculated as the fasting glucose to glycated hemoglobin ratio and was categorized into tertiles (i.e., SHR1–3). In-hospital and 3-month outcomes were compared using multivariable regression models. The impact of SHR stratified by diabetes status was evaluated and the predictive accuracy of the Totaled Health Risks in Vascular Events (THRIVE)-c risk score was explored with the inclusion of SHR. **Results:** Compared to the SHR1–2 groups, the SHR3 group exhibited significantly higher rates of 24 h symptomatic intracranial hemorrhage (adjusted odds ratio [aOR], 4.088; 95% confidence interval [CI], 1.551–10.772; *p* = 0.004) and 72 h early neurological deterioration (aOR, 3.505; 95% CI, 1.984–6.192; *p* < 0.001), while the incidence of post-stroke pneumonia did not differ significantly between the groups (aOR, 1.379; 95% CI, 0.838–2.268; *p* = 0.206). At three months, the SHR3 group had a worse distribution of modified Rankin scale (aOR, 2.261; 95% CI, 1.495–3.421; *p* < 0.001) and faced a higher risk of functional dependence (adjusted hazard ratio [aHR], 1.629; 95% CI, 1.230–2.158; *p* = 0.001) as well as all-cause mortality (aHR, 1.986; 95% CI, 1.235–3.194; *p* = 0.005). The adverse effects of an elevated SHR were more pronounced in non-diabetic patients, and incorporating SHR significantly enhanced the predictive accuracy of the THRIVE-c score for poor stroke outcomes. **Conclusions:** Stress hyperglycemia could be related to the risks of in-hospital complications and 3-month poor outcomes following mechanical thrombectomy in the anterior circulation.

## 1. Background

Stress hyperglycemia is defined as hospital-related hyperglycemia in patients without evidence of previous diabetes or as a deterioration in glycemic control among those with pre-existing diabetes [1]. It is frequently observed in response to acute illness, regardless of diabetes status. In the context of acute ischemic stroke, nearly half of patients (45.2%) were found to experience stress hyperglycemia [2]. To evaluate the impact of stress-induced hyperglycemia on ischemic stroke outcome, various indicators were tested. Among these, the stress hyperglycemia ratio (SHR) has been recommended over the glycemic gap (i.e., absolute increase in glycemia) and over measurements of random or fasting blood glucose levels [3,4]. The SHR accounts for pre-existing diabetes by adjusting for baseline glycemia. It is calculated as the ratio of acute blood glucose to background glucose levels, although no consensual standard exists.

A previous study calculated SHR by dividing fasting blood glucose (FBG) by glycated hemoglobin (HbA1c) [5]. FBG is considered a more reliable glycemic index with minimized dietary influences, while HbA1c reflects background glucose levels over a two- to three-month period. Some studies have used random blood glucose levels at admission to capture the acute changes in glucose during the early stage of stroke [6]. However, SHR calculated using the FBG/HbA1c ratio was found to be significantly and independently associated with poor functional outcomes in patients with acute ischemic stroke, whereas calculations based on admission random blood glucose did not reach significance [4]. In addition, several studies have computed SHR using the estimated average glucose derived from HbA1c with the equation “Estimated average glucose = (1.59 × HbA1c) − 2.59” [7,8,9,10]. According to a recent investigation, this method did not demonstrate better predictive power for stroke outcomes than SHR defined by the FBG/HbA1c ratio [5].

Although mechanical thrombectomy is the first-line treatment for ischemic stroke in the anterior circulation, more than half of treated patients fail to achieve functional independence, and the mortality rate reaches one in four at 90 days [11]. Therefore, a valid prediction model for outcomes following endovascular therapy is crucial for risk stratification. A recent systematic review of 19 prediction models identified the Totaled Health Risks in Vascular Events (THRIVE)-c score as a superior tool in both discrimination and calibration for predicting functional outcomes in patients with anterior circulation, large vessel occlusions undergoing endovascular treatment [12]. The THRIVE-c score was developed on a large cohort [13] and has been externally validated in patients from routine clinical practice [14] and randomized clinical trials [12]. However, it remains uncertain whether incorporating SHR could further improve the predictive ability of THRIVE-c, in light of a prior meta-analysis indicating that a higher SHR is significantly associated with increased rates of poor outcomes, mortality, neurological deficit, hemorrhagic transformation, and infectious complications in stroke patients [15].

Therefore, this study was conducted in acute ischemic stroke patients who underwent endovascular treatment for emergent anterior circulation occlusion. The objectives were to investigate: (1) the impact of different SHR levels on in-hospital outcomes, including symptomatic intracranial hemorrhage (ICH) at 24 h, early neurological deterioration at 72 h, and post-stroke pneumonia; (2) the influence of SHR on functional outcome and all-cause mortality at 3 months; (3) whether these effects differed between patients with and without diabetes; (4) whether integrating SHR into the THRIVE-c prediction model could improve risk stratification for poor outcomes.

## 2. Methods

### 2.1. Study Design

This retrospective observational cohort study screened consecutive patients from May 2017 to December 2021 in the prospective database of the stroke registry at Nanjing First Hospital, Nanjing Medical University, a Demonstration Advanced Stroke Center certified by the China Stroke Prevention Project Committee, National Health Commission [16]. The study was conducted in compliance with the principles of the Declaration of Helsinki and was approved by the research ethics committee of Nanjing First Hospital, Nanjing Medical University, with the approval number 20211011-05. The written informed consent was waived by the committee in view of the retrospective nature of the study, the anonymized evaluation of the registry data, and the procedures being performed as part of the routine clinical care.

### 2.2. Participants

**Figure 1:**
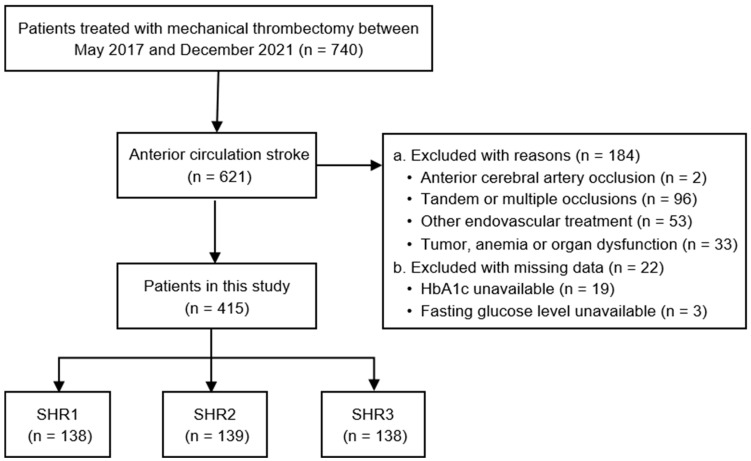
Flow diagram of the study. HbA1c, glycated hemoglobin; SHR, stress hyperglycemia ratio, calculated as fasting serum glucose (mmol/L)/HbA1c (%); SHR1, first SHR tertile (≤0.940); SHR2, second SHR tertile (0.940–1.177); SHR3, third SHR tertile (≥1.177).

The inclusion criteria were as follows: (1) age ≥ 18 years; (2) acute ischemic stroke due to large vessel occlusion in the anterior circulation (i.e., middle cerebral artery, MCA; internal carotid artery, ICA) confirmed by computed tomography angiography or magnetic resonance angiography; (3) receipt of mechanical thrombectomy in accordance with national guidelines. Patients were excluded if they met any of the following criteria: (1) tandem or multiple occlusions; (2) received endovascular treatment other than mechanical thrombectomy (e.g., emergent stent implantation); (3) had a history of malignancy, hemoglobin disorders, or severe cardiac, hepatic, or renal dysfunction; (4) lacked data on fasting blood glucose or HbA1c. A flow diagram of patient selection is provided in Figure 1.

### 2.3. Data on Clinical Characteristics

Baseline demographic and comorbidity data were extracted from the stroke registry. The degree of previous functional disability was assessed using the pre-stroke modified Rankin scale (mRS), with lower mRS values indicating a higher level of premorbid function. The admission blood pressure and stroke severity (assessed by the National Institutes of Health Stroke Scale, NIHSS score) were recorded through face-to-face interviews by neurologists and stroke nurses as part of routine care. Stroke etiology was determined according to the Trial of Org 10,172 in Acute Stroke Treatment (TOAST) criteria. Serum levels of FBG, HbA1c, total cholesterol, triglyceride, low-density lipoprotein cholesterol, blood urea nitrogen (BUN), and estimated glomerular filtration rate (eGFR) were measured within 24 h of admission at the hospital central laboratory by technicians blinded to clinical data. SHR was calculated as FBG (mmol/L)/HbA1c (%) ratio. For further comparisons, patients were stratified into three groups based on tertiles of SHR (i.e., SHR1–3), with a higher SHR indicating more severe stress hyperglycemia [17].

### 2.4. Reperfusion Therapy

Patients eligible for intravenous thrombolysis were treated within a 4.5 h window after symptom onset. The indications for endovascular therapy were a target mismatch profile, defined as an ischemic core volume of <70 mL, a perfusion–diffusion mismatch ratio ≥ 1.8, and a mismatch volume ≥ 15 mL after the year 2018. Before 2018, the procedure was performed within 6 h of witnessed stroke onset. For patients admitted more than 6 h since stroke onset, a perfusion–diffusion mismatch ratio ≥ 1.2, with infarction involving <1/3 of the MCA territory, was required. The endovascular treatment was performed under conscious sedation using stent retrievers, aspiration catheters, or a combination of both, at the discretion of the operator. The door-to-puncture time was recorded. The recanalization status at the end of each procedure was graded using the modified Thrombolysis in Cerebral Infarction scale, with successful recanalization corresponding to a score of 2b to 3. Two experienced neuroradiologists blinded to clinical outcomes reviewed the angiographic data.

### 2.5. Outcomes During Hospitalization and 3-Month Follow-Up

In-hospital outcomes included symptomatic ICH at 24 h, early neurological deterioration within 72 h, and post-stroke pneumonia. Symptomatic ICH was defined as any newly observed ICH, resulting in an increase of ≥4 points in the NIHSS score or any neurological deterioration leading to death [18]. Early neurological deterioration was defined as an NIHSS increase of ≥2 points within 72 h of hospitalization [19]. Post-stroke pneumonia was diagnosed as a lower respiratory tract infection occurring within the first 7 days of hospitalization, based on clinical symptoms, laboratory findings, and confirmed via chest CT [20]. The 3-month follow-up outcomes included functional dependence, defined as an mRS score of 3–6, and all-cause mortality within the period following stroke. The mRS is an ordinal scale used to assess functional disability after stroke, ranging from 0 (no symptoms) to 6 (death) [21], with scores between 3 and 6 indicating functional dependence. The mRS scores were documented in the registry and assessed by well-trained neurologists during scheduled interviews conducted via telephone or face-to-face visits.

### 2.6. Statistical Analysis

Statistical analyses were performed with SPSS (Version 26.0, IBM Corp., Armonk, NY, USA) and R version 4.2.3. Baseline characteristics were compared across the three SHR tertiles. Continuous variables were reported as medians with interquartile ranges (IQR) and compared using the Kruskal–Wallis test. Categorical variables were described as frequencies and percentages, with differences analyzed using the Chi-square test or Fisher’s exact test, as appropriate.

Univariable and multivariable backward stepwise logistic regression analyses were performed to examine the associations between the highest SHR tertile (SHR3) and in-hospital outcomes. The effect estimate was the odds ratio (OR) with 95% confidence interval (CI). The impact of SHR3, compared to SHR1–2, on the distribution of the 3-month mRS scores was evaluated using ordinal shift logistic regression, yielding an adjusted common OR. Comparisons of 3-month follow-up outcomes (i.e., functional dependence and all-cause mortality rate) between SHR3 and SHR1–2 groups were assessed using univariable and multivariable Cox proportional hazards regression models, with the hazard ratio (HR) as the effect estimate. The multivariable models were adjusted for clinically significant factors and covariates with a significant univariate test, including age, sex, hypertension, diabetes mellitus, atrial fibrillation, pre-stroke mRS score, admission systolic blood pressure, baseline NIHSS, BUN, eGFR, successful recanalization, and door-to-puncture time. Fasting blood glucose was excluded as a covariate due to collinearity with SHR tertiles (Spearman correlation coefficient 0.786, *p* < 0.001). Similarly, in-hospital insulin use was not included as a confounder due to collinearity with diabetic history and SHR tertiles (Spearman correlation coefficient 0.486 and 0.271, respectively, both *p* < 0.001).

To assess the predictive value of SHR3 for unfavorable outcomes in stroke patients, receiver operating characteristic (ROC) curve analysis was performed by incorporating either 1 point for SHR3 or continuous SHR into the THRIVE-c risk score. The THRIVE-c score is calculated as the sum of continuous age, continuous NIHSS score, and 1 point each for the history of hypertension, diabetes mellitus, and atrial fibrillation [13]. The areas under the curve (AUCs) were compared between the SHR3-incorporated and conventional THRIVE-c risk scores by the Delong test, and ΔAUC was calculated to evaluate the improvement in risk prediction with SHR3. Statistical significance was set at a two-sided *p* value of <0.05 for all analyses.

## 3. Results

### 3.1. Baseline Characteristics

A total of 415 patients who underwent thrombectomy in the anterior circulation were included, with a median age of 72.0 (62.0–80.0) years, and 38.6% were women. Patients were grouped based on SHR tertiles (Table 1). Baseline characteristics revealed that individuals in the highest SHR tertile were more likely to be older, female, and have a history of atrial fibrillation, whereas other cardiovascular risk factors—including hypertension, diabetes mellitus, and previous stroke or transient ischemic attack—were similar among groups. Compared to the other groups, the SHR3 group demonstrated significantly higher admission systolic blood pressure and NIHSS scores, elevated levels of FBG and BUN, lower eGFR, more frequent in-hospital insulin use, and a lower likelihood of achieving successful recanalization. In contrast, the distribution of HbA1c levels, use of intravenous alteplase and oral hypoglycemics, occlusion site, and door-to-puncture time were comparable among all groups (Table 1).

### 3.2. Clinical Outcomes According to SHR Tertiles

**Figure 2:**
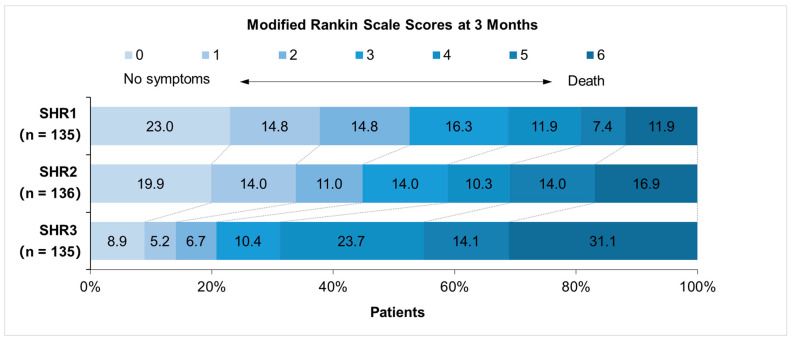
Distribution of modified Rankin scale scores at 3 months stratified by SHR tertiles. SHR, stress hyperglycemia ratio, calculated as fasting serum glucose (mmol/L)/glycated hemoglobin (%); SHR1, first SHR tertile (≤0.940); SHR2, second SHR tertile (0.940–1.177); SHR3, third SHR tertile (≥1.177). Results showing higher SHR tertiles had higher odds of unfavorable outcomes at 3-month follow-up.

Symptomatic ICH was observed in 5.1% of patients at 24 h post-endovascular treatment, with a higher prevalence in the SHR3 group that reached borderline significance across SHR tertiles (Table 1). Early neurological deterioration at 72 h occurred in 19.0% of patients, while post-stroke pneumonia was documented in 62.2% of the cohort; both complications were more frequent in patients within the higher SHG tertiles (Table 1). In multivariable logistic regression analysis, the SHR3 group had a significantly increased risk of symptomatic ICH (adjusted OR, 4.088; 95% CI, 1.551–10.772; *p* = 0.004) and early neurological deterioration (adjusted OR, 3.505; 95% CI, 1.984–6.192; *p* < 0.001) compared with the SHR1–2 groups, while the incidence of post-stroke pneumonia was similar between groups (adjusted OR, 1.379; 95% CI, 0.838–2.268; *p* = 0.206; Table 2). Among the 406 patients (97.8%) with 3-month follow-up data, 246 (60.6%) were functionally dependent, and 82 (20.2%) experienced all-cause mortality. The distribution of mRS scores at 3 months varied across SHR tertiles, with the SHR3 group showing higher odds of unfavorable outcomes (adjusted common OR, 2.261; 95% CI, 1.495–3.421; *p* < 0.001) (Figure 2). Moreover, patients in the highest SHR tertile had a significantly elevated risk of both functional dependence and mortality at the 3-month follow-up (Table 1). Multivariable Cox regression models suggested that the SHR3 group was associated with a 1.629-fold higher risk of functional dependence (adjusted HR, 1.629; 95% CI, 1.230–2.158; *p* = 0.001) and a 1.986-fold higher risk of all-cause mortality (adjusted HR, 1.986; 95% CI, 1.235–3.194; *p* = 0.005) at 3 months compared to the SHR1–2 groups (Table 3).

### 3.3. Impact of Diabetes Status on the Association Between SHR Tertiles and Outcomes

Interestingly, among patients with known diabetes, elevated SHR levels were not associated with a significantly higher risk of early neurological deterioration (adjusted OR, 2.533; 95% CI, 0.810–7.920; *p* = 0.110) or post-stroke pneumonia (adjusted OR, 1.018; 95% CI, 0.424–2.444; *p* = 0.968). Conversely, non-diabetic patients with SHR3 had markedly higher risks of early neurological deterioration (adjusted OR, 5.313; 95% CI, 2.332–12.104; *p* < 0.001) and post-stroke pneumonia (adjusted OR, 4.089; 95% CI, 2.071–8.074; *p* < 0.001). Data on the impact of diabetes status on symptomatic ICH were not available owing to the limited sample size of subgroups. Furthermore, follow-up findings indicated that the association between SHR3 and functional dependence at 3 months reached only borderline significance in diabetic patients (adjusted HR, 1.587; 95% CI, 0.989–2.547; *p* = 0.056) but it was statistically significant in non-diabetic patients (adjusted HR, 1.600; 95% CI, 1.128–2.270; *p* = 0.008). Additionally, significant correlations between SHR3 and 3-month all-cause mortality were consistently observed in both diabetic (adjusted HR, 3.020; 95% CI, 1.219–7.484; *p* = 0.017) and non-diabetic patients (adjusted HR, 1.795; 95% CI, 1.007–3.200; *p* = 0.047).

### 3.4. Added Predictive Value of SHR for Outcomes During Hospitalization and 3-Month Follow-Up

The THRIVE-c score is a simple tool for predicting prognosis after endovascular treatment in stroke patients. In the ROC analysis, the AUC of the THRIVE-c risk score was 0.564 for symptomatic ICH, 0.525 for early neurological deterioration, 0.669 for post-stroke pneumonia, 0.744 for 3-month functional dependence, and 0.690 for all-cause death at three months (Table 4). When SHR was integrated into the risk score—either by assigning 1 point to patients in the highest tertile (SHR3) or by treating it as a continuous variable—it yielded a significant improvement in the AUC compared to the original THRIVE-c score for symptomatic ICH (including SHR3: ∆AUC 0.011; *p* = 0.040; including continuous SHR: ∆AUC 0.072; *p* = 0.020) and early neurological deterioration (including SHR3: ∆AUC 0.005; *p* = 0.001; including continuous SHR: ∆AUC 0.042; *p* < 0.001). In contrast, the improvement for post-stroke pneumonia was not statistically significant (including SHR3: ∆AUC 0.007; *p* = 0.311; including continuous SHR: ∆AUC 0.018; *p* = 0.195). Furthermore, incorporating SHR enhanced the predictive value of the THRIVE-c score for functional dependence (including SHR3: ∆AUC 0.007; *p* = 0.001; including continuous SHR: ∆AUC 0.022; *p* = 0.040) and all-cause mortality (including SHR3: ∆AUC 0.006; *p* = 0.005; including continuous SHR: ∆AUC 0.041; *p* = 0.007) at 3-month follow-up (Table 4).

## 4. Discussion

This study indicated that stroke patients with stress hyperglycemia were more likely to experience poor in-hospital and 3-month outcomes following anterior circulation thrombectomy, and these effects varied according to the premorbid diabetic status. Furthermore, incorporating SHR into the previously validated THRIVE-c risk score improved the stratification of patients at risk of adverse outcomes.

The acute rise in blood glucose, an evolutionarily adaptive response to stress, is mediated by activation of the hypothalamic–pituitary–adrenal axis and the sympathoadrenal system, which increases circulating glucose through enhanced gluconeogenesis, glycogenolysis, and insulin resistance [22]. Although the transient hyperglycemia provides a ready energy source for the brain and immune system [22], it may also promote platelet hyperactivity, coagulopathy, and endothelial dysfunction. These effects can trigger thrombotic and inflammatory processes that exacerbate neurovascular injury [23]. Numerous studies have established stress hyperglycemia as a strong predictor of poor prognosis in acute ischemic stroke [2,3,4,5,6,7,8,9,10,17,24,25,26,27,28,29,30,31,32,33], even among patients treated with first-line intravenous thrombolysis [4,5,6,30] or mechanical thrombectomy [2,7,8,9,17,26,27,28].

The present study explored the relationship between stress hyperglycemia and in-hospital adverse events in patients undergoing anterior circulation thrombectomy. We found that patients in the SHR3 group were over four times more likely to develop symptomatic ICH and 3.5 times more likely to suffer early neurological deterioration. This finding is consistent with recent reports that link stress hyperglycemia to an increased risk of symptomatic ICH in stroke patients admitted within seven days of onset [32], in those treated with endovascular therapy in the anterior [26,28] and posterior [9] circulation, as well as in patients receiving intravenous thrombolysis [5]. Similarly, the relationship between SHR and early neurological deterioration has been observed in strokes resulting from large vessel occlusion [27] and single subcortical infarction [10]. Our data also showed a trend towards a higher incidence of post-stroke pneumonia with higher SHR tertiles, yet this relationship did not reach statistical significance after adjustment, even though a prior study reported an independent association between SHR and post-stroke pneumonia [31].

Next, this study revealed that patients in the highest SHR tertile had markedly poorer 3-month recovery: they were twice as likely to shift to a worse mRS score, faced a 63% higher risk of functional dependence, and had a nearly doubled risk of death. In line with our results, prior investigations have highlighted SHR as a risk factor for poor 3-month mRS outcomes in patients undergoing mechanical thrombectomy in both the anterior [2,7,17,28] and posterior [9,26,27] circulation, as well as in those treated with thrombolysis [4] or presenting with single subcortical infarction [10]. Moreover, severe stress hyperglycemia significantly increased risks of 3-month all-cause mortality in this cohort, which aligns with recent studies implying that SHR can be a valid marker for 3-month mortality prediction following mechanical thrombectomy [8,26,28]. However, the predictive role of SHR in patients treated with intravenous thrombolysis remains controversial, warranting further investigation [4,5].

Both non-diabetic and diabetic patients experiencing an acute stroke may develop stress hyperglycemia, with incidences ranging from 8% to 63% in non-diabetics, and from 39% to 83% in diabetics [34]. Notably, our data showed that non-diabetic patients with stress hyperglycemia suffer worse outcomes following anterior circulation thrombectomy. Likewise, in patients with vertebrobasilar artery occlusion who received endovascular treatment, only the non-diabetic subgroup showed a significant association between SHR and adverse post-stroke outcomes [9]. One plausible explanation is that non-diabetic patients lack the chronic adaptations to high glucose levels [23]. Unlike diabetic patients, they may mount a more pronounced inflammatory response during acute hyperglycemia—a phenomenon observed in conditions such as acute myocardial infarction [35] and trauma [36], where non-diabetic individuals consistently exhibited higher levels of inflammatory cytokines (e.g., IL-6). Furthermore, a recent in vitro study demonstrated that, when exposed to a high-glucose environment, non-diabetic endothelium showed greater susceptibility in terms of angiogenic ability, whereas the pre-existing microangiopathy in diabetics may offset the additional negative effects of stress hyperglycemia [37]. Furthermore, non-diabetic patients are less likely to benefit from glucose-lowering therapies aimed at mitigating hyperglycemia-induced damage, while certain anti-diabetic medications (e.g., pioglitazone) may offer protective effects against stroke [38]. Nonetheless, one study found no interaction between SHR and diabetes status in predicting 3-month functional recovery and all-cause mortality after thrombectomy for anterior circulation stroke [28]. Others argue that stress hyperglycemia is an epiphenomenon of the most severe strokes and it is the severity itself driving poor outcomes [39]. Therefore, prospective studies are needed to clarify the interactive effects of comorbid diabetes and SHR on stroke prognosis and to assess whether a history of diabetes confers any neuroprotective benefit in the setting of post-stroke stress hyperglycemia.

The THRIVE-c risk score is a recently validated tool for predicting outcomes after acute ischemic stroke, with free web calculators available at http://www.mdcalc.com/thrive-score-for-stroke-outcome/ (accessed on 26 March 2025) [12]. In our cohort, incorporating SHR into the THRIVE-c score improved the stratification of patients with in-hospital and 3-month poor outcomes, suggesting the potential to yield a more accurate prognostic tool, pending further validation in larger populations to ensure reliability and generalizability. The detrimental effects of stress hyperglycemia in stroke patients may be attributed to several mechanisms. First, the aggravated risk of hemorrhagic transformation in patients with elevated SHR may be related to mitochondrial dysfunction [40] and oxidative stress [41] in endothelial cells, resulting in endothelial dysfunction, hyperpermeability, apoptosis, and inflammatory infiltration [32]. Second, the increased risk of early neurological deterioration in stroke patients with stress hyperglycemia may result from exacerbated anaerobic metabolism, neurovascular injury, and thrombo-inflammation [23], which promote infarct growth, collateral failure, and hemorrhagic transformation [27]. Finally, the effect of SHR on 3-month outcomes may be attributable to the shared mechanisms underlying in-hospital complications and the causative role stress hyperglycemia might have played in atherosclerotic plaque progression [42], leading to a higher risk of subsequent stroke despite dual antiplatelet therapy [43], thereby reducing the likelihood of favorable outcomes and increasing mortality risk at follow-up.

Several limitations should be considered in the interpretation of our findings. First, the retrospective study design may be subject to selection bias, and certain potential confounders (body mass index, insulin resistance, and inflammatory biomarkers) were not measured. Hence, our results need replication in settings with prospective designs. Second, the follow-up period was limited to three months after the event. However, this is the most critical period for functional recovery. Further long-term follow-up studies are required to verify our results. Third, we only focused on SHR upon admission and it cannot capture acute glycemic fluctuations, so it remains to be determined whether dynamic changes in glucose levels (e.g., a 24 h glycemic profile) or follow-up SHR measurements impact stroke prognosis. Fourth, the protective effects of prior anti-diabetic drug use against stress hyperglycemia could not be assessed due to incomplete medication history data. Finally, given the exploratory nature of our observational study and the conflicting evidence regarding strict glucose control [44], the optimal management of stress hyperglycemia and target glycemic ranges remain to be elucidated.

## 5. Conclusions

This study adds to the growing body of evidence that stress hyperglycemia could be associated with poor outcomes in the setting of ischemic stroke. Incorporating SHR into the THRIVE-c risk score could improve predictive accuracy for adverse events during hospitalization and at 3-month follow-up in stroke patients receiving mechanical thrombectomy in the anterior circulation. Further studies are required to develop tailored interventions for SHR management and deepen the mechanistic understanding of its role in functional recovery among both non-diabetic and diabetic stroke patients.

## Figures and Tables

**Table 1:** Baseline characteristics according to different SHR tertiles.

Characteristics	SHR Tertiles			*p*
	SHR1 (≤0.940)	SHR2 (0.940–1.177)	SHR3 (≥1.177)	
Patient no.	138	139	138	
Demography				
Age (years)	69.0 (60.0–76.0)	71.0 (61.0–80.0)	76.0 (67.0–82.0)	<0.001
Sex (female)	48 (34.8%)	46 (33.1%)	66 (47.8%)	0.023
Comorbidities				
Hypertension	99 (71.7%)	108 (77.7%)	98 (71.0%)	0.384
Diabetes mellitus	42 (30.4)	37 (26.6%)	42 (30.4%)	0.722
Atrial fibrillation	50 (36.2%)	49 (35.3%)	70 (50.7%)	0.014
Previous stroke/TIA	38 (27.5%)	33 (23.7%)	30 (21.7%)	0.522
Admission metrics				
Pre-stroke mRS score ≤ 2	134 (97.1%)	133 (96.4%) (*n* = 138)	132 (96.4%) (*n* = 137)	0.926
Admission SBP	133 (120–150)	140 (123–157)	144 (127–159)	0.022
Admission DBP	83 (76–93)	86 (76–95)	86 (74–98)	0.477
Admission NIHSS score	13 (8–17)	13 (10–18)	16 (12–19)	<0.001
TOAST				0.140
Large artery atherosclerosis	65 (47.1%)	68 (48.9%)	50 (36.2%)	
Cardioembolism	59 (42.8%)	55 (39.6%)	75 (54.3%)	
Others	14 (10.1%)	16 (11.5%)	13 (9.4%)	
Blood test				
FBG (mmol/L)	4.8 (4.3–5.4)	6.4 (5.7–6.9)	8.5 (7.4–10.2)	<0.001
HbA1c	5.9 (5.6–6.8)	5.9 (5.5–6.4)	5.9 (5.5–6.6)	0.495
Total cholesterol (mmol/L)	4.0 (3.4–4.9)	4.1 (3.4–4.9) (*n* = 138)	4.1 (3.5–4.9) (*n* = 136)	0.627
Triglyceride (mmol/L)	1.0 (0.7–1.4)	1.1 (0.8–1.5) (*n* = 138)	1.0 (0.7–1.5) (*n* = 136)	0.217
LDL	2.4 (1.9–3.1)	2.4 (2.0–3.1) (*n* = 138)	2.4 (1.9–3.0) (*n* = 136)	0.944
BUN (mmol/L)	4.8 (4.3–5.4)	6.4 (5.7–6.9)	8.5 (7.4–10.2)	0.005
eGFR (mL/(min·1.73 m^2^))	78.7 (59.9–98.9)	75.9 (55.4–105.6)	65.4 (51.0–88.7)	0.002
In-hospital medications				
Intravenous alteplase	50 (36.2%)	54 (38.8%)	63 (45.7%)	0.257
Insulin use	38 (22.6%)	47 (28.0%)	83 (49.4%)	<0.001
Oral hypoglycemics	22 (33.8%)	20 (30.8%)	23 (35.4%)	0.867
Occlusion site				0.496
Intracranial ICA	42 (30.4%)	48 (34.5%)	54 (39.1%)	
The first segment of MCA	87 (63.0%)	81 (58.3%)	72 (52.2%)	
The second segment of MCA	9 (6.5%)	10 (7.2%)	12 (8.7%)	
Mechanical thrombectomy procedure				
Door-to-puncture time	115.0 (88.0–145.0) (*n* = 135)	110.0 (85.0–135.0) (*n* = 135)	113.0 (85.0–148.3) (*n* = 134)	0.819
Successful recanalization	124 (89.9%)	126 (90.6%)	111 (80.4%)	0.019
Adverse outcomes				
Symptomatic ICH	5 (3.6%)	4 (2.9%)	12 (8.7%)	0.056
Early neurological deterioration	18 (22.8%)	19 (24.1%)	42 (53.2%)	<0.001
Post-stroke pneumonia	70 (50.7%)	89 (64.0%)	99 (71.7%)	0.001
3-month functional dependence	64 (47.4%) (*n* = 135)	75 (55.1%) (*n* = 136)	107 (79.3%) (*n* = 135)	<0.001
3-month all-cause mortality	16 (11.9%) (*n* = 135)	23 (16.9%) (*n* = 136)	43 (31.9%) (*n* = 135)	<0.001

SHR, stress hyperglycemia ratio, calculated as fasting serum glucose (mmol/L)/HbA1c (%); SHR1, first SHR tertile (≤0.940); SHR2, second SHR tertile (0.940–1.177); SHR3, third SHR tertile (≥1.177); TIA, transient ischemic attack; mRS, modified Rankin scale; SBP, systolic blood pressure; DBP, diastolic blood pressure; NIHSS, National Institute of Neurological Stroke Scale; TOAST, trial of Org 10,172 in acute stroke treatment; FBG, fasting blood glucose; HbA1c, glycosylated hemoglobin; LDL, low-density lipoprotein cholesterol; BUN, blood urea nitrogen; eGFR, estimated glomerular filtration rate; ICA, internal carotid artery; MCA, middle cerebral artery; ICH, intracranial hemorrhage.

**Table 2:** Associations between SHR3 and in-hospital clinical outcomes.

In-Hospital Outcomes	SHR1–2	SHR3	*p*	Univariable Analysis		Multivariable Analysis *	
				OR (95% CI)	*p*	OR (95% CI)	*p*
Total population	*n* = 277	*n* = 138					
Symptomatic ICH	9 (3.2%)	12 (8.7%)	0.017	2.836 (1.165–6.905)	0.022	4.088 (1.551–10.772)	0.004
Early neurological eterioration	37 (13.4%)	42 (30.4%)	<0.001	2.838 (1.719–4.685)	<0.001	3.505 (1.984–6.192)	<0.001
Post-stroke pneumonia	159 (57.4%)	99 (71.7%)	0.005	1.884 (1.213–2.927)	0.005	1.379 (0.838–2.268)	0.206
Diabetic patients	*n* = 79	*n* = 42					
Symptomatic ICH	2 (2.5%)	5 (11.9%)	0.048				
Early neurological deterioration	10 (12.7%)	10 (23.8%)	0.116	2.156 (0.816–5.697)	0.121	2.533 (0.810–7.920)	0.110
Post-stroke pneumonia	49 (62.0%)	27 (64.3%)	0.807	1.102 (0.506–2.399)	0.807	1.018 (0.424–2.444)	0.968
Non-diabetic patients	*n* = 198	*n* = 96					
Symptomatic ICH	7 (3.5%)	7 (7.3%)	0.240				
Early neurological deterioration	27 (13.6%)	32 (33.3%)	<0.001	3.167 (1.760–5.697)	<0.001	5.313 (2.332–12.104)	<0.001
Post-stroke pneumonia	110 (55.6%)	72 (75.0%)	0.001	2.400 (1.398–4.120)	0.001	4.089 (2.071–8.074)	<0.001

SHR, stress hyperglycemia ratio, calculated as fasting serum glucose (mmol/L)/HbA1c (%); SHR1–2, first and second SHR tertiles (≤1.177); SHR3, third SHR tertile (≥1.177); OR, odds ratio; CI, confidence interval; ICH, intracranial hemorrhage. * Adjusted for age, sex, hypertension, diabetes mellitus, atrial fibrillation, pre-stroke mRS score, admission systolic blood pressure, baseline NIHSS, blood urea nitrogen, estimated glomerular filtration rate, successful recanalization, and door-to-puncture time.

**Table 3:** Associations between SHR3 and 3-month clinical outcomes.

3-Month Outcomes	SHR1–2	SHR3	*p*	Univariable Analysis		Multivariable Analysis *	
				HR (95% CI)	*p*	HR (95% CI)	*p*
Total population	*n* = 271	*n* = 135					
Functional dependence	139 (51.3%)	107 (79.3%)	<0.001	1.799 (1.393–2.324)	<0.001	1.629 (1.230–2.158)	0.001
All-cause mortality	39 (14.4%)	43 (31.9%)	<0.001	2.460 (1.573–3.848)	<0.001	1.986 (1.235–3.194)	0.005
Diabetic patients	*n* = 75	*n* = 42					
Functional dependence	49 (65.3%)	32 (76.2%)	0.222	1.518 (0.962–2.395)	0.073	1.587 (0.989–2.547)	0.056
All-cause mortality	11 (14.7%)	13 (31.0%)	0.036	2.492 (1.076–5.770)	0.033	3.020 (1.219–7.484)	0.017
Non-diabetic patients	*n* = 196	*n* = 93					
Functional dependence	90 (45.9%)	75 (80.6%)	<0.001	1.937 (1.419–2.645)	<0.001	1.600 (1.128–2.270)	0.008
All-cause mortality	28 (14.3%)	30 (32.3%)	<0.001	2.346 (1.376–4.001)	0.002	1.795 (1.007–3.200)	0.047

SHR, stress hyperglycemia ratio, calculated as fasting serum glucose (mmol/L)/HbA1c (%); SHR1–2, first and second SHR tertiles (≤1.177); SHR3, third SHR tertile (≥1.177); HR, hazard ratio; CI, confidence interval. * Adjusted for age, sex, hypertension, diabetes mellitus, atrial fibrillation, pre-stroke mRS score, admission systolic blood pressure, baseline NIHSS, blood urea nitrogen, estimated glomerular filtration rate, successful recanalization, and door-to-puncture time.

**Table 4:** The added predictive value of SHR on THRIVE-c risk score for stroke outcomes.

	AUC (95% CI)	ΔAUC	*p*
Symptomatic ICH at 24 h			
THRIVE-c	0.564 (0.465–0.663)	-	-
THRIVE-c + SHR3	0.575 (0.460–0.690)	0.011	0.040
THRIVE-c + SHR-c	0.636 (0.510–0.763)	0.072	0.020
Early neurological deterioration at 72 h			
THRIVE-c	0.525 (0.453–0.596)	-	-
THRIVE-c + SHR3	0.530 (0.462–0.598)	0.005	0.001
THRIVE-c + SHR-c	0.572 (0.497–0.678)	0.042	<0.001
Pneumonia within 7 d of admission			
THRIVE-c	0.669 (0.617–0.721)	-	-
THRIVE-c + SHR3	0.676 (0.622–0.729)	0.007	0.311
THRIVE-c + SHR-c	0.687 (0.635–0.740)	0.018	0.195
3-month functional dependence			
THRIVE-c	0.744 (0.697–0.791)	-	-
THRIVE-c + SHR3	0.751 (0.703–0.799)	0.007	0.001
THRIVE-c + SHR-c	0.766 (0.720–0.813)	0.022	0.040
3-month all-cause mortality			
THRIVE-c	0.690 (0.631–0.750)	-	-
THRIVE-c + SHR3	0.696 (0.635–0.758)	0.006	0.005
THRIVE-c + SHR-c	0.731 (0.671–0.792)	0.041	0.007

DeLong’s test for two correlated ROC curves. SHR, stress hyperglycemia ratio, calculated as fasting serum glucose (mmol/L)/HbA1c (%); THRIVE-c, the Totaled Health Risks in Vascular Events (THRIVE)-c risk score; THRIVE-c + SHR3, defined as THRIVE-c score plus 1 additional point for patients in the highest SHR tertile (≥1.177); THRIVE-c + SHR-c, defined as incorporating SHR as a continuous variable into the THRIVE-c score; AUC, area under the curve; ICH, intracranial hemorrhage.

## Data Availability

The data presented in this study are available on request from the corresponding author due to privacy reasons.

## List of Abbreviations

AUCs	areas under the curve
BUN	blood urea nitrogen
CI	confidence interval
eGFR	estimated glomerular filtration rate
FBG	fasting blood glucose
HbA1c	glycated hemoglobin
HR	hazard ratio
ICA	internal carotid artery
ICH	intracranial hemorrhage
IQR	interquartile ranges
OR	odds ratio
MCA	middle cerebral artery
mRS	modified Rankin scale
NIHSS	the National Institutes of Health Stroke Scale
ROC	receiver operating characteristic
SHR	stress hyperglycemia ratio
THRIVE	Totaled Health Risks in Vascular Events
TOAST	the Trial of Org 10,172 in Acute Stroke Treatment
